# Supplementary material for: Antibiotic Use and Resistance Knowledge Assessment of Personnel on Chicken Farms with High Levels of Antimicrobial Resistance: A Cross-Sectional Survey in Ica, Peru
Source: Antibiotics (Basel). 2022 Feb 1;11(2):190. doi: 10.3390/antibiotics11020190 (PMC8868202; doi:10.3390/antibiotics11020190)
Supplement: Supplementary file 1 [file antibiotics-11-00190-s001.zip › S1 Survey English.pdf]

ANTIBIOTICS RESISTANCE QUESTIONNAIRE (date ....../....../...........)

AGE (years): **GENDER:** ☐ Female ☐ Male

EDUCATION (year of study): ☐ Early childhood education ☐ Primary ☐ Secondary

1) When did you last take antibiotics?

☐ In the last month

☐ In the last 6 months

☐ In the last year

☐ More than a year ago

☐ Never

☐ I can't remember

If "Never" have chosen, go directly to question 4

2) On that occasion, did you get the antibiotics (or a prescription for them) from a doctor or nurse?

☐ Yes

☐ No

☐ I can't remember

3) On that occasion, where did you get the antibiotics?

☐ Medical store or pharmacy

☐ Stall or hawker

☐ The internet

☐ Friend or family member

☐ I had them saved up from a previous time

☐ Somewhere/someone else

☐ Can't remember

4) When do you think you should you stop taking antibiotics once you have begun treatment?

☐ When I feel better

☐ When I have taken all of the antibiotics as directed

☐ I don't know

5) Do you think this statement is 'true' or 'false'? "It's okay to use antibiotics that were given to a friend or family member, as long as they were used to treat the same illness"

☐ True

☐ False

☐ Don't know

6) Do you think this statement is 'true' or 'false'? "It's okay to buy the same antibiotics, or request these from a doctor, if you're sick and they helped you get better when you had the same symptoms before"

☐ True
☐ False
☐ Don't know

7) Do you think these conditions can be treated with antibiotics?

☐ HIV/AIDS
☐ Gonorrhoea
☐ Bladder infection or urinary tract infection (UTI)
☐ Diarrhoea
☐ Cold and flu
☐ Fever
☐ Malaria
☐ Measles
☐ Skin or wound infection
☐ Sore throat
☐ Body aches
☐ Headaches

8) Have you heard of any of the following terms

☐ Antibiotic resistance
☐ Superbugs
☐ Antimicrobial resistance
☐ AMR
☐ Drug resistance
☐ Antibiotic-resistant bacteria

9) On the scale shown, how much do you agree the following actions would help address the problem of antibiotic resistance?

		True	False
1	People should use antibiotics only when they are prescribed by a doctor or nurse		
2	Farmers should give fewer antibiotics to food-producing animals		
3	People should not keep antibiotics and use them later for other illnesses		
4	Parents should make sure all of their children's vaccinations are up-to-date		
5	People should wash their hands regularly		
6	Doctors should only prescribe antibiotics when they are needed		
7	Governments should reward the development of new antibiotics		
8	Pharmaceutical companies should develop new antibiotics		
