# Supplementary material for: Antibiotic Use and Resistance Knowledge Assessment of Personnel on Chicken Farms with High Levels of Antimicrobial Resistance: A Cross-Sectional Survey in Ica, Peru
Source: Antibiotics (Basel). 2022 Feb 1;11(2):190. doi: 10.3390/antibiotics11020190 (PMC8868202; doi:10.3390/antibiotics11020190)
Supplement: Supplementary file 1 [file antibiotics-11-00190-s001.zip › S2 Survey Spanish.pdf]

CUESTIONARIO SOBRE RESISTENCIA ANTIBIÓTICA (fecha ....../....../...........)

EDAD (años): **GÉNERO:** ☐ Femenino ☐ Masculino

EDUCACIÓN (nivel de estudios): ☐ Inicial ☐ Primaria ☐ Secundaria

1) ¿Cuándo tomó antibióticos por última vez?

☐ En el último mes
☐ En los últimos 6 meses
☐ En el último año
☐ Hace más de un año
☐ Nunca
☐ No recuerda

Si eligió "Nunca", ir directamente a la pregunta 5

2) En esa ocasión, ¿obtuvo los antibióticos de un doctor o enfermero (a)?

☐ Sí
☐ No
☐ No recuerda

3) En esa ocasión, ¿de dónde obtuvo los antibióticos?

☐ Botica o farmacia
☐ Puesto o vendedor ambulante
☐ Internet
☐ Amigo o familiar
☐ Los tenía guardados de una vez anterior
☐ Otro lugar/ alguien más
☐ No recuerda

4) ¿Cuándo considera que debe dejar de tomar antibióticos una vez iniciado el tratamiento?

☐ Cuando me sienta mejor
☐ Cuando haya tomado todos los antibióticos según lo indicado
☐ No sabe

5) ¿Considera la siguiente afirmación ser 'verdadera' o 'falsa'? "Está bien usar antibióticos que fueron provistos por un amigo o familiar, siempre y cuando fueron usados para tratar la misma enfermedad"

☐ Verdadero
☐ Falso
☐ No sabe

6) ¿Considera la siguiente afirmación ser 'verdadera' o 'falsa'? "Está bien comprar los mismos antibióticos, o solicitarlos a un doctor, si está enfermo y anteriormente ayudaron a hacerlo sentir mejor cuando tuvo los mismos síntomas"

☐ Verdadero

☐ Falso

☐ No sabe

7) ¿Cuáles de estas condiciones considera pueden ser tratadas con antibióticos?

☐ VIH/SIDA

☐ Gonorrea

☐ Infección de las vías urinarias (ITU) o cistitis

☐ Diarrea

☐ Resfrío o gripe

☐ Fiebre

☐ Malaria

☐ Sarampión

☐ Infección de piel o herida

☐ Dolor de garganta

☐ Dolor de cuerpo

☐ Dolor de cabeza

8) ¿Cuáles de los siguientes términos ha escuchado?

☐ Resistencia antibiótica

☐ Superbacteria

☐ Resistencia antimicrobiana

☐ AMR

☐ Resistencia a las drogas

☐ Bacteria resistente a antibióticos

10) En la escala, ¿cuánto está de acuerdo con las siguientes acciones que ayudarían a abordar el problema de la resistencia a los antibióticos?

		Verdadero	Falso
1	Las personas deberían usar antibióticos solo cuando reciban una prescripción de un doctor o enfermera		
2	Los granjeros deberían dar menos antibióticos a los animales de producción		
3	Las personas no deberían guardar antibióticos y usarlos posteriormente para otras enfermedades		
4	Los padres deberían asegurarse que sus hijos tengan todas sus vacunas al día		
5	Las personas deberían lavarse las manos regularmente		
6	Los doctores solo deberían prescribir antibióticos cuando es necesario		
7	Los gobiernos deberían recompensar el desarrollo de nuevos antibióticos		
8	Las compañías farmacéuticas deberían desarrollar nuevos antibióticas		
